# Supplementary material for: A scalable and cGMP-compatible autologous organotypic cell therapy for Dystrophic Epidermolysis Bullosa
Source: Nat Commun. 2024 Jul 11;15:5834. doi: 10.1038/s41467-024-49400-z (PMC11239819; doi:10.1038/s41467-024-49400-z)
Supplement: Supplementary file 6 — Supplementary Data 3 [file 41467_2024_49400_MOESM6_ESM.zip › Supplementary Data 3/57_offtarget_sites/CO265B_2KB/patient2.chr11_60159330_60159352.2KB.pdf]

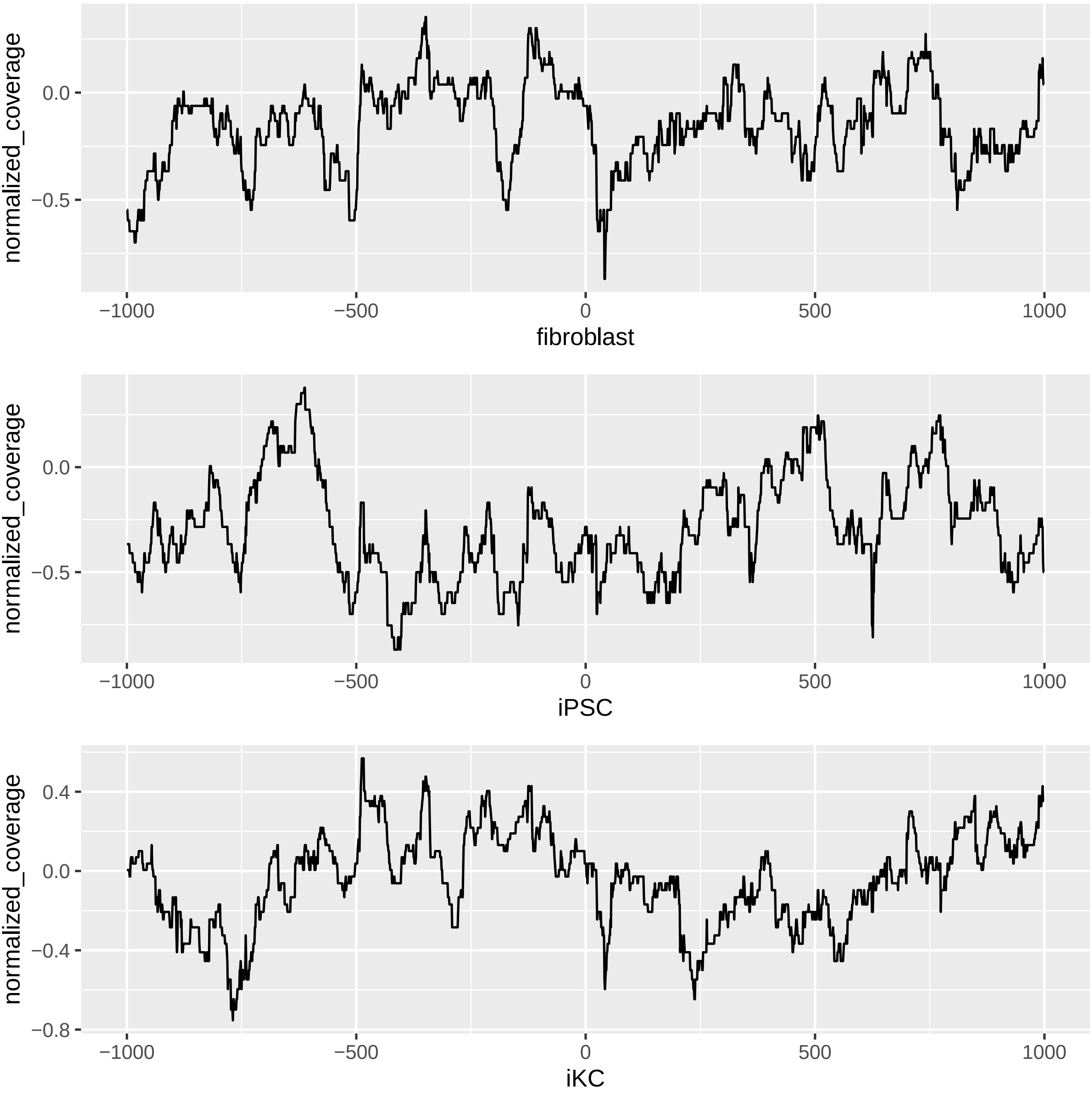
normalized_coverage
0.0
−0.5
−1000
−500
0
500
1000
fibroblast
normalized_coverage
0.0
−0.5
−1000
−500
0
500
1000
iPSC
normalized_coverage
0.4
0.0
−0.4
−0.8
−1000
−500
0
500
1000
iKC
